# Cytochemical Localization of Polysaccharides in *Dendrobium officinale* and the Involvement of *DoCSLA6* in the Synthesis of Mannan Polysaccharides

**DOI:** 10.3389/fpls.2017.00173

**Published:** 2017-02-14

**Authors:** Chunmei He, Kunlin Wu, Jianxia Zhang, Xuncheng Liu, Songjun Zeng, Zhenming Yu, Xinghua Zhang, Jaime A. Teixeira da Silva, Rufang Deng, Jianwen Tan, Jianping Luo, Jun Duan

**Affiliations:** ^1^Key Laboratory of South China Agricultural Plant Molecular Analysis, Gene Improvement, South China Botanical Garden, Chinese Academy of SciencesGuangzhou, China; ^2^Independent ResearcherMiki-cho, Japan; ^3^School of Food Science and Engineering, Hefei University of TechnologyHefei, China

**Keywords:** PAS, histology, polysaccharide granules, *CSLA*, *Dendrobium officinale*

## Abstract

*Dendrobium officinale* is a precious traditional Chinese medicinal plant because of its abundant polysaccharides found in stems. We determined the composition of water-soluble polysaccharides and starch content in *D*. *officinale* stems. The extracted water-soluble polysaccharide content was as high as 35% (w/w). Analysis of the composition of monosaccharides showed that the water-soluble polysaccharides were dominated by mannose, to a lesser extent glucose, and a small amount of galactose, in a molar ratio of 223:48:1. Although starch was also found, its content was less than 10%. This result indicated that the major polysaccharides in *D*. *officinale* stems were non-starch polysaccharides, which might be mannan polysaccharides. The polysaccharides formed granules and were stored in plastids similar to starch grains, were localized in *D*. *officinale* stems by semi-thin and ultrathin sections. *CELLULOSE SYNTHASE-LIKE A* (*CSLA*) family members encode mannan synthases that catalyze the formation of mannan polysaccharides. To determine whether the *CSLA* gene from *D*. *officinale* was responsible for the synthesis of mannan polysaccharides, *35S*:*DoCSLA6* transgenic lines were generated and characterized. Our results suggest that the *CSLA* family genes from *D. officinale* play an important role in the biosynthesis of mannan polysaccharides.

## Introduction

*Dendrobium*, with approximately 1450 species, is the third largest genus of the *Orchidaceae* (Zhang et al., 2016). Many bioactive constituents have been identified from *Dendrobium* species and polysaccharides are regarded as the main bioactive substances, displaying immunomodulatory and hepatoprotective activities (Ng et al., 2012).

Polysaccharides, including starch and non-starch polysaccharides, are the main constituent of plant biomass and the major energy source in the human diet (Choct, 1997; Sorek et al., 2014). Non-starch polysaccharides, which not only have a cell wall, but also serve as energy storage material, can be classified into three main groups: cellulose, non-cellulosic polymers, and pectic polysaccharides (Bailey, 1973; Choct, 1997; Sinha et al., 2011). Mannan polysaccharides are a sub-group of non-cellulosic polymers that play an important role in higher plants. For example, they act as structural elements in the cell wall to maintain cell integrity (Schröder et al., 2009; Wang et al., 2012), serve as reserve polysaccharides in the walls of seed endosperm and vacuoles in vegetative tissues to feed cells and adjust osmotic potential (Meier and Reid, 1982). In addition, mannan polysaccharides have beneficial effects on human health such as increasing cytokine expression (Hsieh et al., 2008), and have excellent antioxidant and anticancer activity (Joseph et al., 2013).

The mannan family is a major constituent of hemicellulose that is widespread in plant tissues, and can be divided into four subfamilies: linear mannan, glucomannan, galactomannan (GM), and galactoglucomannan (GGM; Choct, 1997; Petkowicz et al., 2001; Moreira, 2008). Pure mannans are artificially defined as polysaccharides that contain more than 90% of mannose sugar residues (Meier and Reid, 1982; Buckeridge, 2010). In higher plants, pure mannans are widespread in the cell wall of seeds, such as *Schizolobium amazonicum*, *Coffea arabica* beans, and *Carum carvi asa* (Hopf and Kandler, 1977; Navarini et al., 1999; Petkowicz et al., 2001). In addition, pure mannan is also found in the *Orchidaceae*, such as *Oncidium* (cv. ‘Gower Ramsey’; Wang et al., 2006). Glucomannans found in seeds contain a β-(1,4)-linked D-mannose backbone and β-(1,4)-linked D-glucose residues at different ratios (Ishrud et al., 2001), and exist widely in the vegetative tissues of members of the *Liliaceae*, *Araceae* and *Orchidaceae* (Meier and Reid, 1982). *Amorphophallus konjac* (*Araceae*) contains a slightly acetylated glucomannan (konjac mannan) that has been thoroughly studied (Nishinari et al., 1992). Some orchids contain glucomannans with a partially acetylated structure, similar to that of konjac mannan (Hua et al., 2004; Hsieh et al., 2008; Xing et al., 2014, 2015). GMs with a β-(1,4)-linked D-mannose backbone and a α-(1,6)-linked galactose side chain serve as reserve hemicellulosic polysaccharides that are deposited in the cell wall of the seed endosperm and fruit rind (Reid et al., 1987; Joseph et al., 2013). GGMs, which serve as structural polysaccharides in secondary cell walls, consist of β-(1,4)-D-mannopyranosyl and β-(1,4)-D-glucopyranosyl residues with single α-(1,6)-D-galactopyranosyl units at the *O*-6 position of D-mannose or D-glucose residues (Capek et al., 2000). They have been widely characterized in many plant species and are present throughout the plant kingdom (Lišková et al., 2006). GGMs have been found in red clover (*Trifolium pratense*; Buchala and Meier, 1973), kiwifruit (*Actinidia deliciosa*; Schröder et al., 2001), tobacco (*Nicotiana tabacum*; Eda et al., 1984, 1985), Norway spruce (*Picea abies*; Capek et al., 2000; Willför et al., 2003; Polari et al., 2012) and Arabidopsis (*Arabidopsis thaliana*, Voiniciuc et al., 2015).

The biosynthesis of mannan polysaccharides is catalyzed by mannan synthases, which use GDP-D-mannose or GDP-D-glucose as their substrate (Hassid, 1969). Mannan synthases have been isolated from many higher plant species such as the seeds of fenugreek (*Trigonella foenum*-*graecum* L.) and guar (*Cyamopsis tetragonoloba*), which contain abundant GM in their seed endosperms (Edwards et al., 1989), as well as in pea seedlings (*Pisum sativum*; Piro et al., 1993) and senna (*Senna occidentalis*; Reid et al., 1995). To date, studies have showed that the *CELLULOSE SYNTHASE-LIKE A (CSLA)* genes from *Cyamopsis tetragonoloba* and *A. thaliana* encode mannan synthases and play an important role in the synthesis of mannan polysaccharides (Dhugga et al., 2004; Liepman et al., 2005; Goubet et al., 2009; Yu et al., 2014). In higher plants, the cellulose synthase-like (*CSL*) families (*CSLA*-*H* and *CSLJ*) belonging to the cellulose synthase (*CESA*) superfamily have been proposed to encode the enzymes involved in the synthesis of non-cellulosic polysaccharides (Richmond and Somerville, 2000; Hazen et al., 2002).

*Dendrobium officinale* contains abundant polysaccharides in the stem and is regarded as a prized folk medicine for its bioactive polysaccharides (Ng et al., 2012; Xing et al., 2014). The major polysaccharide in *D*. *officinale* is glucomannan, accounting for 58.3% of the dry weight (DW) of the crude polysaccharide fraction (Xing et al., 2014). There is little understanding of the storage and localization of *D*. *officinale* polysaccharides in stems because previous studies focused mainly on their extraction and structural characterization. In this study, to extend our understanding of the localization of polysaccharides in *D*. *officinale*, we carried out a histochemical analysis of polysaccharides using periodic acid–Schiff (PAS) stain and ultramicroscopic observations of *D*. *officinale* stems. In addition, eight *CSLA* genes (*DoCSLA*1-*8*), which had been identified from changes in mannose content throughout four developmental stages (He et al., 2015), were analyzed to provide genetic evidence for their involvement in mannan synthesis. This work, which is important to understand the localization of polysaccharides in *D*. *officinale* and the molecular mechanisms controlling the biosynthesis of mannan polysaccharides in this orchid, will shed new light on the localization of non-starch polysaccharides.

## Materials and Methods

### Plant Materials and Growth Conditions

*Dendrobium officinale* plants, potted in a substrate of shattered fir bark, were maintained in a greenhouse in the South China Botanical Garden, Guangzhou, China under natural conditions. About 13-month-old plants which sprouted in April were used to determine water-soluble polysaccharide, monosaccharide and starch content, as well as the localization of polysaccharides.

In this study, *A. thaliana* ecotype Columbia (sustained in our laboratory) plants served as the wild type (WT) and were used for transgenic experiments. Plants were grown in a growth chamber under a 16-h photoperiod (100 μmol m^-2^ s^-1^) at 22°C. To screen transgenic plants, seeds were sown and germinated on Murashige and Skoog (1962) medium with 1.5% (w/v) sucrose and 0.8% (w/v) agar, and supplemented with 30 mg/L hygromycin B. Plants were potted in a substrate of topsoil and vermiculite (1:3, v/v), and periodically watered with liquid Hyponex fertilizer (N:P:K = 6-10-5, diluted 1,000-fold; Hydroponic Chemicals Co., Findlay, OH, USA).

### Water-Soluble Polysaccharide Content and Analysis of Monosaccharides

Stems from about 13-month-old *D*. *officinale* were harvested (two stems from each pot, and at least 100 pots), cleaned, and dried in an oven at 105°C until constant weight. Samples were powdered to a fine powder by a DFT-50 pulverizer (Xinno Instrument Equipment Inc., Shanghai, China) and used to analyze water-soluble polysaccharide content and monosaccharide composition. To extract the water-soluble polysaccharides, the powder (0.3 g) was pre-extracted in 80% ethanol for 2 h at 80°C and filtered through Whatman filter paper No. 1. The residue was extracted with double-distilled water for 2.5 h at 100°C. Double-distilled water was added to the supernatant and made up to 250 mL after the residue was filtered out by Whatman filter paper No. 1. This stock was deemed as the polysaccharide solution and was used for the analysis of water-soluble polysaccharide content by the phenol-sulfuric acid method according to Dubois et al. (1956) and He et al. (2015). Briefly, 200 μL of polysaccharide solution was mixed with 1800 μL of double-distilled water, added 1 mL of 5% phenol and rapidly vortexed, then mixed with 5 mL of concentrated sulfuric acid. The reaction solution was placed in a 100°C bath for 20 min. The absorbance of the sample solution was measured at 488 nm with a UV-6000 spectrophotometer (Shanghai Metash, Shanghai, China) when the reaction solution had cooled down to room temperature. The reaction solution, when added to 2000 μL of distilled water, was used as the calibration standard. Glucose was used to calculate a standard curve (10, 20, 40, 60, 80, and 100 μg/mL). Each sample was assayed as three replicates.

For the analysis of monosaccharides from *D*. *officinale* stems, 0.12 g of powder described above of each sample was used to extract water-soluble polysaccharides that were analyzed by high performance liquid chromatography (HPLC) according to The State Pharmacopoeia Commission of People’s Republic of China (2010) and He et al. (2015). Briefly, the powder of each sample was pre-extracted with 80% ethanol at 80°C for 2 h. This process was repeated four times to remove monosaccharides, oligosaccharides and ethanol-soluble materials, then water-soluble polysaccharides were extracted with double-distilled water at 100°C for 2.5 h. The extraction was hydrolyzed by 3.0 M HCl, derivatized with 1-phenyl-3-methyl-5-pyrazolone (PMP) and monosaccharide content was analyzed by HPLC according to He et al. (2015).

For the analysis of mannose from *A. thaliana*, the above-ground parts (leaves, flowers, and stems) from 2-month-old *A*. *thaliana* ecotype Columbia and transgenic lines were harvested, cleaned and grounded to a fine powder with liquid nitrogen using a mortar and pestle, then dried in an oven at 80°C until constant weight. To analysis mannose content, 0.3 g of powder was pre-extracted with 80% ethanol for 2 h, then extracted with double-distilled water for 4 h at 100°C. Four volumes of 100% ethanol were added to the extracted solution, mixed and kept at 4°C overnight, then centrifuged at 10,000 rpm for 20 min. The residue was re-dissolved in 20 mL of double-distilled water to form the polysaccharide solution. This polysaccharide solution was hydrolyzed and derivatized, and the mannose content was analyzed by HPLC, as described above.

### Extraction and Determination of Starch

The powdered samples used in the analysis of mono- and polysaccharides were also used to determine starch content. Starch extraction and determination were performed according to McCready et al. (1950). Briefly, 0.200 g of powdered sample was wet with a few drops of 80% alcohol in a 50 mL centrifuge tube, 5 mL distilled water was added followed by 25 mL of 80% ethanol. This mixture was vortexed thoroughly with a vortex mixer (Scilogex, Berlin, NH, USA). After left to stand at room temperature for 5 min, the mixture was centrifuged by a universal 32R (Hettich, Tuttlingen, Germany) at 2,500 rpm for 5 min. The residue was pre-extracted by 30 mL of hot 80% ethanol until a test with anthrone (Morris, 1948) proved negative. To extract starch, 5 mL of distilled water and 30 mL of 52% perchloric acid were added to a centrifuge tube that contained the residue described above, and vortexed thoroughly by a Scilogex vortex mixer for 10 min and centrifuged by universal 32R at 2,500 rpm for 10 min. The supernatant was collected into a 100 mL volumetric flask. The extraction was repeated and the supernatant was collected into a volumetric flask. The combined solutions were diluted to 100 mL, filtered through Whatman filter paper No. 1, and the first 5 mL of the solution was discarded. The starch solution was diluted so that it contained 20 to 100 μg of starch per 1 mL. Starch solution (2 mL) was transferred to a 10 mL test tube, 6 mL of anthrone-sulfuric acid solution (2 g of anthrone per 1 L of 95% sulfuric acid) was added, vortexed thoroughly, cooled in water for 2 min and placed in a 100°C bath for 5 min. The absorbance of the sample solution was measured at 630 nm with a UV-6000 spectrophotometer (Shanghai Metash, Shanghai, China) after cooling to room temperature. The reaction solution, which was added to 2 mL of distilled water, replaced the starch solution and was used as the calibration standard. Each sample was assayed as three replicates.

### Histological and Histochemical Analysis and Localization of Polysaccharides in *D*. *officinale* Stems

The stems from 13-month-old *D*. *officinale* were cut into 5 mm long transects and fixed in a solution of 2.5% glutaraldehyde and 2% paraformaldehyde in 0.1 M sodium phosphate buffer (pH 7.2). Samples were cut longitudinally to 4–8 mm^2^ cross-sections under a SZX7 stereoscopic microscope (Olympus America Inc., Center Valley, PA, USA) and immersed in the same fixative while cutting. Segments were collected into sampling bottles filled with fixative then vacuum infiltrated for at least 30 min to facilitate penetration of the fixative and then kept at 4°C for about 7 days. After fixation, samples were washed six times with 1% sodium phosphate buffer, 30 min each time, and post-fixed in 1% osmium tetroxide (OsO_4_) in 0.1 M sodium cacodylate buffer for 4 h (pH 7.2). A graded series of ethanol (30, 50, 75, 85, 95, 100%, v/v) was used to wash and dehydrate samples for 30 min in each step. For osmosis, segments were treated in a graded series buffer (acetone: Epon812, 3:1, 1:1, 1:3) for 30 min in each step, then immersed in Epon812 overnight. On the second day, segments were placed in embedding molds (Beijing Zhongjingkeyi Technology Co., Ltd., Beijing, China) with Epon812 and baked in an oven at 60°C for 2 days. Serial cross-sections of embedded material were cut to 1 μm thickness with an LKB-11800 ultramicrotome (LKB, Bromma, Sweden) and the PAS reaction was performed to stain sections, as described by Tütüncü Konyar et al. (2013). Cross-sections were photographed with a Leica S8 APO stereomicroscope (Leica Microsystems Ltd., Heerbrugg, Switzerland). For transmission electron microscope observations, materials were cut into ultrathin sections (50–70 nm) by a Leica-EM-UC6 ultramicrotome (Leica Microsystems GmbH, Wetzlar, Germany), and then examined and photographed with a JEOL-JEM-1010 transmission electron microscope (Jeol Ltd., Tokyo, Japan) at 100 kV.

### Phylogenetic Analysis

Eight *DoCSLA*s that were likely involved in the biosynthesis of mannan polysaccharides were identified in our previous study (He et al., 2015). To comprehensively analyze the evolutionary relationships of the *CLSA* family between *D*. *officinale* and other plant species, amino acid sequences of CSLA proteins from a dicot (*A. thaliana*) and a monocot (*Oryza sativa* L.) were used to construct an unrooted tree with the Neighbor-Joining method (Saitou and Nei, 1987).

### Gene Structure Analysis

The genomic sequences of these *DoCSLA*s were downloaded from whole genome assemblies of *D. officinale* (DDBJ/EMBL/GenBank accession code: JSDN00000000, Zhang et al., 2016). Genomic and mRNA sequences were used as queries to generate a gene structure diagram with the Gene Structure Display Server^1^ (Hu et al., 2015). The motifs in the amino acid sequences of DoCSLAs were identified using MEME 4.11.2^2^.

### Construction of *35S*:*DoCSLA6* Vector and Transformation in *Arabidopsis thaliana*

Total RNA was extracted from *D*. *officinale* stems with Column Plant RNAout2.0 (Tiandz, Inc., Beijing, China) and reverse transcribed for first-strand cDNA by using M-MLV reverse transcriptase (Promega, Madison, WI, USA) according to the manufacturer’s protocol. The cDNA was used as a template to amplify the *DoCSLA6* gene with a specific set of primers (DoCSLA6OxF/DoCSLA6OxR; Supplementary Table 1) and cloned into the *Nco*I site of the binary vector pCAMBIA-1302 by an In-Fusion^®^ HD Cloning Kit (Takara Bio Inc., Dalian, China) according to the manufacturer’s instructions. Expression of the *DoCSLA6* gene was under the control of the *CaMV35S* promoter. The *35S*:*DoCSLA6* construct was introduced into *Arabidopsis* plants (ecotype Col) by an *Agrobacterium*-mediated (*Agrobacterium tumefaciens*, EHA105 strain) method described by Clough and Bent (1998).

### Semi-Quantitative RT-PCR Analysis to Assess the Expression Levels of *DoCSLA6* in Arabidopsis

Leaves from about 1-month-old *Arabidopsis* plants were collected and kept in liquid nitrogen to extract total RNA. The TRIzol RNA isolation method (TRIzol, Invitrogen, Carlsbad, CA, USA) was used for total RNA extraction as described by Meng and Feldman (2010). Two microgram of total RNA was used for reverse-transcription reactions by the M-MLV Reverse Transcriptase Kit (Promega) according to the manufacturer’s protocol. For the PCR reaction, the Taq DNA Polymerase Kit (Takara Bio Inc.) was used with the following amplification protocol: 94°C for 3 min; 30 cycles (25 cycles for *AtUBQ10*) of 94°C for 30 s, 55°C for 30 s, 72°C for 1 min; a final elongation step at 72°C for 10 min. PCR products (5 μL) were evaluated by electrophoresis on a 1% agarose gel in TAE buffer and photographed with Gel Documentation System GenoSens 1880 (Shanghai Qinxiang Scientific Instrument Co. Ltd., Shanghai, China). The *A. thaliana ubiquitin10* gene (*AtUBQ10*) was used as an internal control based on the recommendation of Zhao et al. (2015). The primers (DoCSLA6F/DoCSLA6R and AtUBQ10F/AtUBQ10R) used for qRT-PCR are listed in Supplementary Table 1.

### Statistical Analyses

Data were analyzed using SigmaPlot12.3 software (Systat Software Inc., San Jose, CA, USA) by a *t*-test. *P* < 0.05 was considered to be statistically significant.

## Results

### Mannose-Containing Polysaccharides Are the Main Polysaccharides in the Stems of *D. officinale*

Generally, starch is the major type of storage polysaccharide in higher plant species. To understand the type of polysaccharides in *D*. *officinale* stems, water-soluble polysaccharides, the monosaccharide fraction of water-soluble polysaccharides, and starch were analyzed. Water-soluble polysaccharides were abundant, about 367 mg/g, in the stems of *D*. *officinale* (**Table 1**). The main monosaccharide within the water-soluble polysaccharides was mannose, about 257 mg/g (**Table 1**), indicating that the main polysaccharides were mannan polysaccharides. Glucose was the second most common monosaccharide, found at 55 mg/g (**Table 1**) in the water-soluble polysaccharide fraction. Galactose was also found in the water-soluble polysaccharide fraction, but it had a very low content, about 1 mg/g (**Table 1**). Although starch is perceived to be the major type of non-structural polysaccharide in plants, the starch content in *D*. *officinale* stems was about 93 mg/g. These results indicate that mannan polysaccharides are the main polysaccharides in *D*. *officinale* stems, in agreement with Xing et al. (2014) and Wei et al. (2016).

**Table 1:** Water-soluble polysaccharides content and monosaccharide composition of water-soluble polysaccharides in stems of *D. officinale* (mg/g DW).

Water-soluble polysaccharides	Mannose	Glucose	Galactose
366.79 ± 8.30	256.58 ± 0.50	55.12 ± 0.19	1.15 ± 0.03

*The water-soluble polysaccharides of monosaccharides are as determined by high performance liquid chromatography (HPLC) analysis of 1-phenyl-3-methyl-5-pyrazolone (PMP) derivatives. All experiments consist of three independent replicates. DW, dry weight.*

### Stem Anatomy and Localization of Polysaccharides

**FIGURE 1:**
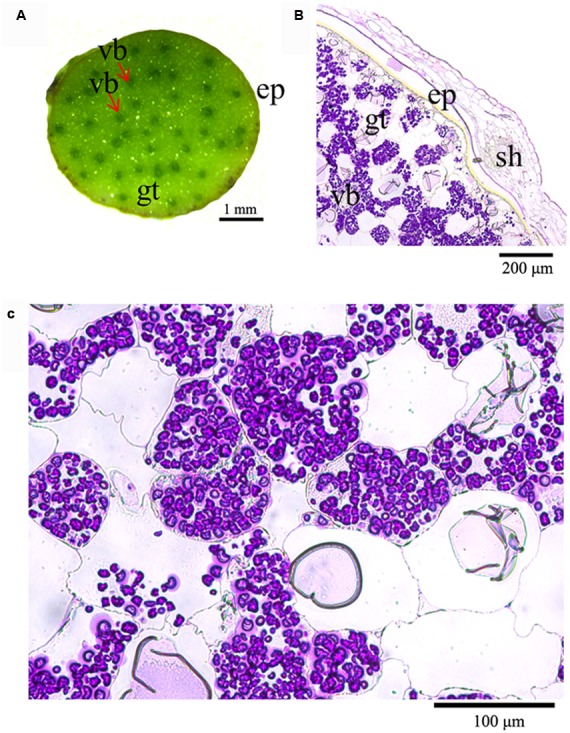
**Histochemical analysis of the location of polysaccharides in *D*. *officinale* stems. (A)** Cross section of a *D*. *officinale* stem. **(B)** Detection of polysaccharides in *D*. *officinale* stems by the periodic acid–Schiff (PAS) method. **(C)** Enlarged view of **(B)** ep, epidermis; gt, ground tissue; vb, vascular bundle; sh, sheath.

Having understood that the stems of *D*. *officinale* contain a high content of mannan polysaccharides and a low starch content, we investigated the localization of polysaccharides in stems by anatomical observations. Transverse sections from fresh stems showed that a number of vascular bundles were dispersed throughout the stem, similar to other monocots (**Figure 1A**). Ground tissue was composed of a mass of parenchyma cells among which vascular bundles were embedded (**Figures 1A,B**). The ground tissue stained with PAS was strongly labeled, but weak labeling of the sheath and a weak signal in the walls of cortical cells and parenchyma cells (**Figure 1B**). Surprisingly, polysaccharides formed granules in parenchyma cells stained an intense purple with polysaccharide stains (**Figures 1B,C**).

### Polysaccharide Granules Localized in Plastids

**FIGURE 2:**
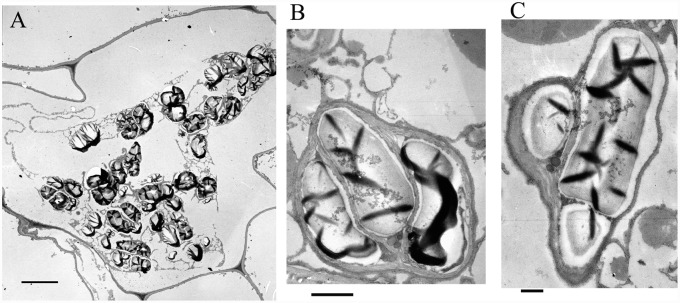
**Electron microscopic observations on the polysaccharide granules. (A)** Morphology of cells containing polysaccharide granules; bar = 10 μm. **(B)** A plastid containing polysaccharide granules; bar = 1 μm. **(C)** A plastid containing polysaccharide granules; bar = 500 nm.

In order to identify the localization of polysaccharide granules at the subcellular (organelle) level, ultrathin sections were made and analyzed. There was no discernible nucleus, vacuole or cellular organelles in parenchyma cells, but numerous polysaccharide granules were clearly visible (**Figure 2A**). A complicated membrane system, in which the polysaccharide granules were embedded, was present in parenchyma cells (**Figure 2A**). The polysaccharide granules had various forms with unequal size and were localized in plastids (**Figures 2B,C**). The membrane structure of plastids was clearly visible, and wrapped several polysaccharide granules in a single plasmid (**Figures 2B,C**). The stems contained a considerable amount of polysaccharides that were stored in the plastids.

### Phylogenetic Analysis of DoCSLA Proteins in *D*. *officinale*, *A. thaliana*, and Rice

**FIGURE 3:**
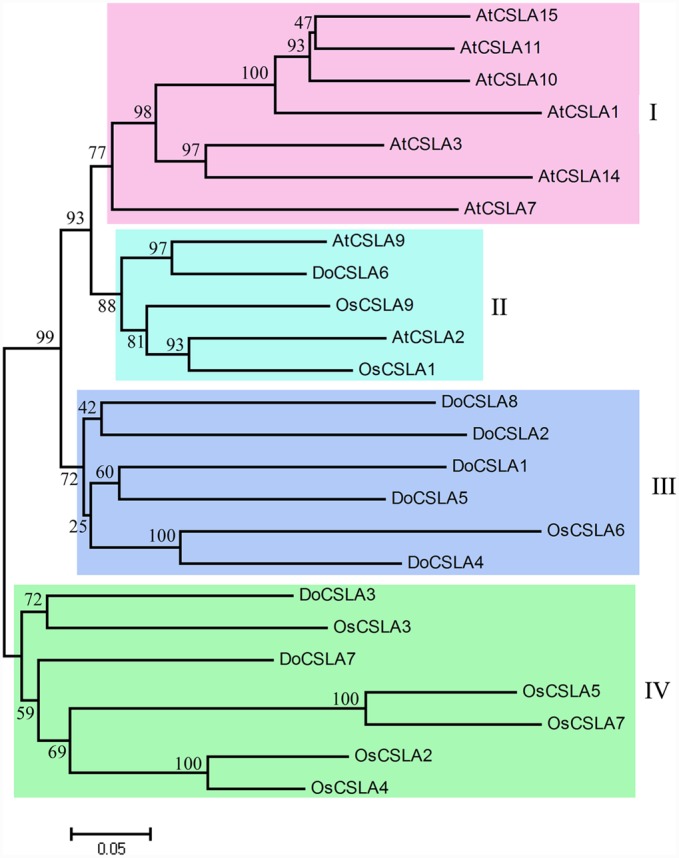
**Molecular phylogenetic tree of the amino acid sequences of the CSLA family of *Arabidopsis thaliana*, *Oryza sativa* (Japonica Group) and eight DoCSLA proteins from *D. officinale*.** The tree was constructed using MEGA 4 by the neighbor-joining method. Protein sequences used for alignment are as follows: AtCSLA1, AAO42230.1; AtCSLA10, NP_173818.1; AtCSLA11, NP_197123.2; AtCSLA14, NP_191159.2; AtCSLA15, NP_193077.2; AtCSLA2, BAB11680.1; AtCSLA3, AAN15522.1; AtCSLA7, AAL24081.1; AtCSLA9, CAB82941.1; OsCslA1, XP_015625335; OsCslA3, XP_015644248; OsCslA4, XP_015630733; OsCslA5, XP_015627865; OsCslA6, Q6Z2T9; OsCslA7, BAC79726; OsCslA9, XP_015643705; *DoCSLA1*, KM980199; *DoCSLA2*, KM980200; *DoCSLA3*, KP003920; *DoCSLA4*, KM980201; *DoCSLA5*, KM980202; *DoCSLA6*, KF195561, *DoCSLA7*, KP205040; *DoCSLA8*, KP205041.

Mannan polysaccharides were the main polysaccharides in *D*. *officinale* stems, accounting for about 58.3% of crude polysaccharides. In the phylogenetic tree, the CSLA family was divided into two branches: clusters I–III in one branch and cluster IV in another branch, indicating that two ancestral genes were the origins of CSLA in both dicots and monocots (**Figure 3**). In addition, the phylogenetic tree clearly showed that CSLA members were separated into four clusters: cluster I included only *A. thaliana*; cluster II included proteins of both the dicot (*A. thaliana*) and monocot (rice); clusters III and IV included proteins from *D*. *officinale* and rice but not from *Arabidopsis* (**Figure 3**). *DoCSLA6* was included in cluster II, and had a close relationship with cluster I (**Figure 3**).

### Analysis of Gene Structure and Motifs in DoCSLA Proteins

**FIGURE 4:**
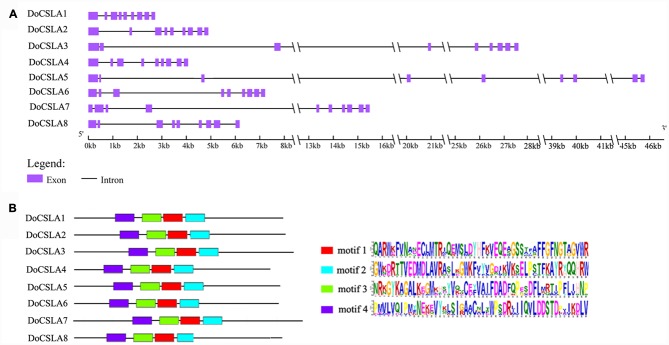
**Analysis of gene structure of *DoCSLA* genes and motifs of DoCSLA proteins. (A)** The gene structure of *DoCSLA* genes. Gene structure analysis was conducted online (http://gsds.cbi.pku.edu.cn/index.php). **(B)** Diagram of the key motifs in the amino acid sequences of DoCSLA proteins. Motif analysis was performed using Meme 4.11.2 software as described in the methods. The sequences of key motifs (motif l–4) are shown on the bottom right of the figure.

Most of the *CSLA*s possess nine exons and eight introns as was observed in rice and *Arabidopsis* (Richmond and Somerville, 2000; Hazen et al., 2002). In order to gain information about the gene structure of *DoCSLA*s, genomic regions of *D*. *officinale* corresponding to *DoCSLA*s were identified and used to analyze the architecture of introns and exons. Most members shared similar intron/exon structures but the length of their genomic region differed (**Figure 4A**). Most *DoCSLA*s (excluding *DoCSLA7*) contained nine exons and eight introns similar to other *CSLA* family members (**Figure 4A**). However, *DoSCLA7* showed variation in intron/exon organization, and contained 10 exons and nine introns (**Figure 4A**). The length of the genomic region was also different. For example, the genomic region of *DoCSLA1* was no longer than 3 kb, but *DoCSLA3*, *DoCSLA5* and *DoCSLA7* were longer than 15 kb (**Figure 4A**).

To better understand the similarity and diversity of motifs in the protein sequences of DoCSLAs, the conserved motifs in proteins were investigated. Among the 12 distinct conserved motifs identified in all of the DoCSLAs, motifs 1–4 collectively comprised the catalytic subunit (**Figure 4B**).

### *DoCSLA6* Contribute to the Mannose Content of Water-Soluble Polysaccharides

**FIGURE 5:**
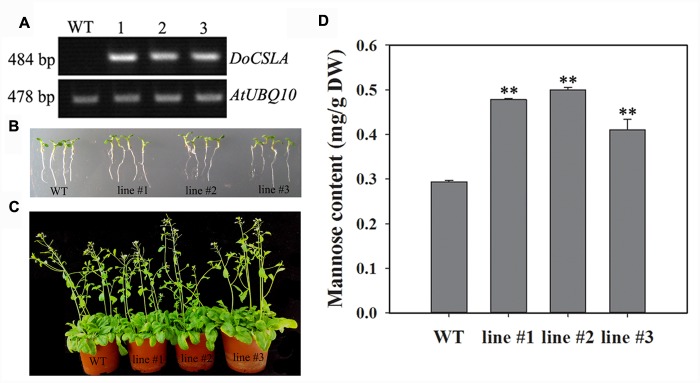
**The phenotypes and mannose content in WT and *35S*:*DoCSLA6* transgenic lines. (A)** Analysis of *DoCSLA6* expression in WT and transgenic lines by semi-quantitative RT-PCR. **(B)** Seedlings of WT and transgenic lines about 1 week old showed no obvious phenotypic changes. Seedlings grew on MS medium with 1.5% (w/v) sucrose and 0.8% (w/v) agar in a 16-h photoperiod (100 μmol m^-2^ s^-1^) at 22°C. **(C)** WT and transgenic lines (about 6 weeks old) showed no obvious differences. WT and transgenic lines potted in a substrate in a 16-h photoperiod (100 μmol m^-2^ s^-1^) at 22°C. **(D)** Mannose content increased in *A. thaliana* expressing the *DoCSLA6* gene under the control of the CaMV 35S promoter. WT, wild-type plant; *35S*:*DoCSLA6* transgenic lines: line #1, line #2, and line #3. Each data bar represents the mean ± standard deviation (SD; *n* = 3). ^∗∗^*P* < 0.01 between WT and transgenic lines by *t*-test. DW, dry weight.

Only four out of nine *CSLA* genes, namely *AtCSLA2*, *AtCSLA3*, *AtCSLA7*, and *AtCSLA9*, are known to produce mannan polysaccharides in *A*. *thaliana* (Sandhu et al., 2009; Dhugga, 2012). A phylogenetic tree analysis showed that *DoCSLA6* had a close relationship with *AtCSLA9* and *AtCSLA2*, and may play a similar role to these genes. Consequently, over-expression (OE) lines of *DoCSLA6* were generated and analyzed. The transcription of *DoCSLA6* was detected in the OE lines but not in the WT plant, suggesting that the *DoCSLA6* gene were successfully transformed with a normal transcript in *A. thaliana* (**Figure 5A**). The HPLC-UV chromatograms are shown in **Supplementary Figure 1**. The OE lines showed no distinct phenotype compared with WT plants (**Figures 5B,C**). However, the mannose content was significantly higher in the OE lines with 0.478, 0.4997, and 0.4105 mg/g DW in lines #1, #2, and #3, respectively, while the WT plant only contained 0.294 mg/g DW (**Figure 5D**). This result suggests that *DoCSLA6* contributed to the synthesis of mannan polysaccharides.

## Discussion

Mannan polysaccharides are important carbohydrates and have at least two functions, as structural constituents of cell walls, and as storage materials during plant growth and development (Pauly and Keegstra, 2008; Wang et al., 2012). They are widely found in plant species such as *A. thaliana*, *Amorphophallus konjac*, *Aloe vera*, and *Populus tremula* and occur in several organs such as roots, leaves, flowers and seeds (Handford et al., 2003; Hamman, 2008; Gille et al., 2011; Wang et al., 2012). Mannan polysaccharides are also found in orchids. For example, a pure mannan polysaccharide was extracted from the pseudobulbs of *Oncidium* (Wang et al., 2006), and glucomannans were found in *Dendrobium* species such as *D*. *huoshanense* and *D*. *officinale* (Hsieh et al., 2008; Xing et al., 2015). Water-soluble polysaccharides in *D*. *officinale* stems contained a low amount of glucose and a high mannose content. This result suggests that mannan polysaccharides are the major type of water-soluble polysaccharides in *D*. *officinale*. Xing et al. (2014) demonstrated that *O*-acetyl-glucomannan with a molar ratio (mannose: glucose) of 6.9:1 was the major polysaccharide in *D*. *officinale*. Even though galactose was detected in the water-soluble polysaccharides of *D*. *officinale*, the molar amount was difficult to estimate.

Polysaccharides have two main biological functions, as reserve materials and as structural components in plants. *D*. *officinale* mannan polysaccharides occur in stems as reserve substances. The PAS method could detect polysaccharides including starch and non-starch polysaccharides. Although starch was also found in *D*. *officinale*, the content only accounted for 93 mg/g DW in *D*. *officinale* stems, suggesting that most of the polysaccharide granules that stained purple were not starch but more likely mannan polysaccharides. In previous studies, mannan polysaccharides in vegetative tissues were only found in roots, tubers, and bulbs where they acted as reserve substances (Meier and Reid, 1982). Obviously, the mannan polysaccharides in the stems of *D*. *officinale* serve as storage materials rather than structural polysaccharides. In addition, starch was the principal reserve polysaccharide and was considered to be the only polysaccharide that formed in the plastids of higher plants (Meier and Reid, 1982). The non-starch polysaccharides in *D*. *officinale* stems formed as granules localized in plastids, similar to starch grains (**Figures 1** and **2**).

Among the *CSL* families, members of the *CSLA* family were the most abundant when compared with other *CSL* families, with nine genes found in the *A. thaliana* and rice genomes (Richmond and Somerville, 2000; Hazen et al., 2002). A recent study showed that 13 proteins encoded by *CSLA* genes showed homology to the *CSLA* family (Zhang et al., 2016). The first functional proof that CSLA proteins are responsible for the synthesis of mannan polysaccharides was from a study on guar (*Cyamopsis tetragonoloba*), which accumulated GM in more than 90% of the endosperm at maturity (Dhugga et al., 2004). *CSLA* plays an important role in the synthesis of mannan polysaccharides, and its function is conserved across different plant species. *CSLA* in *A. thaliana*, *Amorphophallus konjac* and *Populus trichocarpa* encode synthases that participate in the synthesis of β-1,4-mannan polysaccharides *in vitro* (Liepman et al., 2005; Suzuki et al., 2006; Gille et al., 2011). The mannose content increased when *DoCSLA6* was over-expressed in *A. thaliana*, indicating that *DoCSLA6* contributed to the synthesis of mannan polysaccharides. Although mutation of *atcsla9* caused a substantial reduction in glucomannan accumulation in stems, the *atcsla9* mutants displayed no obvious phenotype under laboratory conditions (Goubet et al., 2009). Moreover, the reduction of glucomannan in the *csla2csla3csla9* triple mutant caused no alteration in stem strength and showed similar plant growth and development as WT *A. thaliana* (Goubet et al., 2009). In this study, the phenotype of OE lines with a high mannose content grew similar to laboratory-grown WT plants, indicating that constitutive expression of *DoCSLA6* had a similar function as *AtCSLA*.

## Conclusion

*Dendrobium officinale*, a precious traditional Chinese herb, contains abundant polysaccharides in its stems. These polysaccharides are mainly composed of mannan polysaccharides. A large number of polysaccharide granules, which were found in parenchyma cells, were stored in plastids. *DoCSLA6* is responsible for the production of mannose and may serve as mannan synthase involved in the synthesis of mannan polysaccharides. This study will help orchid biotechnologists understand the localization and synthesis of *D*. *officinale* polysaccharides.

## Author Contributions

JD supervised the project. CH and KW conceived the research and designed the experiments. JZ, XL, and XZ generated transgenic lines. ZY measured starch content and analyzed the monosaccharide composition. CH and RD conducted semithin and ultrathin sections. CH, JAT, JT, SZ, and JL collectively interpreted the results and wrote all drafts of the manuscript. All authors approved the final draft for submission and take full public responsibility for the content of the manuscript.

## Conflict of Interest Statement

The authors declare that the research was conducted in the absence of any commercial or financial relationships that could be construed as a potential conflict of interest.

## Footnotes

1 http://gsds.cbi.pku.edu.cn/index.php

2 http://meme-suite.org/

## Supplementary Material

FIGURE S1 **HPLC-UV chromatograms of *Arabidopsis thaliana*. (A)** HPLC-UV chromatograms of WT. **(B–D)** HPLC-UV chromatograms of line #1–line #3, respectively. WT, wild-type plant; *35S*:*DoCSLA6* transgenic lines: line #1, line #2 and line #3; PMP, 1-phenyl-3-methyl-5-pyrazolone; Man, mannose.
