# Supplementary figures and images for: Cytochemical Localization of Polysaccharides in Dendrobium officinale and the Involvement of DoCSLA6 in the Synthesis of Mannan Polysaccharides
Source: Front Plant Sci. 2017 Feb 14;8:173. doi: 10.3389/fpls.2017.00173 (PMC5306395; doi:10.3389/fpls.2017.00173)

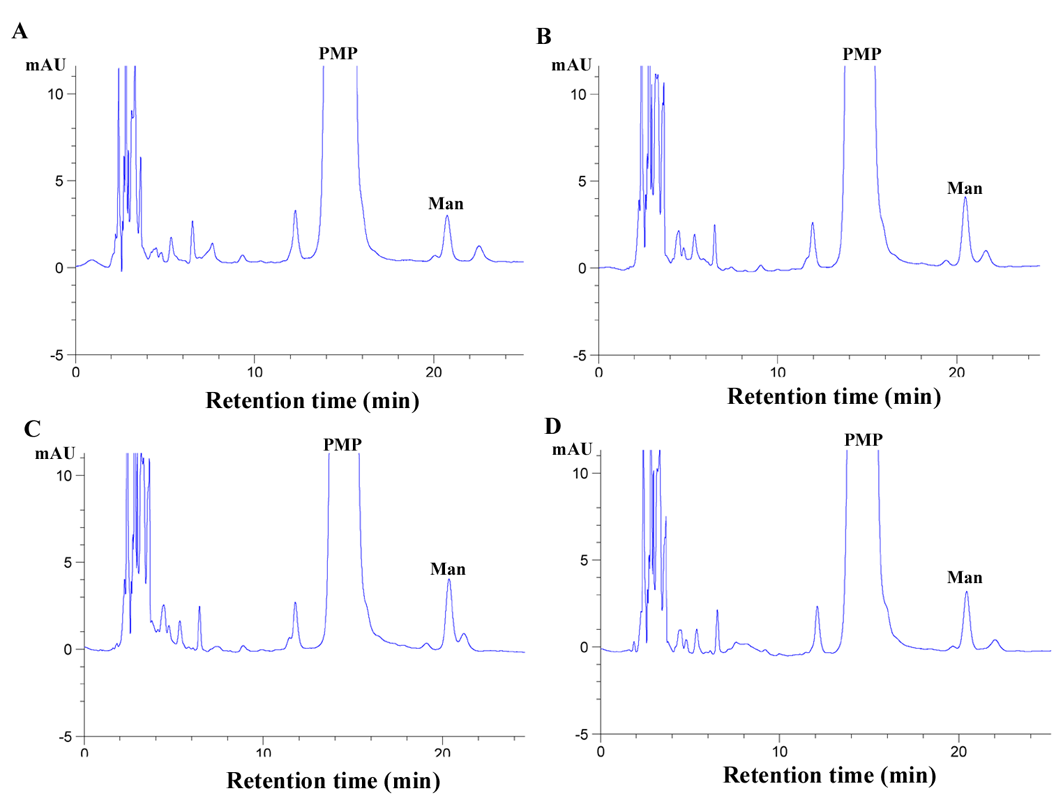

Supplement: FIGURE S1 — HPLC-UV chromatograms of Arabidopsis thaliana. (A) HPLC-UV chromatograms of WT. (B–D) HPLC-UV chromatograms of line #1–line #3, respectively. WT, wild-type plant; 35S:DoCSLA6 transgenic lines: line #1, line #2 and line #3; PMP, 1-phenyl-3-methyl-5-pyrazolone; Man, mannose. [file Image_1.TIF]
