# Supplementary material for: Cytochemical Localization of Polysaccharides in Dendrobium officinale and the Involvement of DoCSLA6 in the Synthesis of Mannan Polysaccharides
Source: Front Plant Sci. 2017 Feb 14;8:173. doi: 10.3389/fpls.2017.00173 (PMC5306395; doi:10.3389/fpls.2017.00173)
Supplement: Supplementary file 2 [file Table_1.pdf]

Supplementary Table 1 Primers used for vector construction and semi-quantitative RT-PCR

Primer name	Primer sequence
DoCSLA6OxF	5′- GGACTCTTGACCATGGATCGAGCAATGGAGAGCGTGAC -3′
DoCSAL6OxR	5′- GTCAGATCTACCATGGTGGAGTGAGGAACAAATATGCCAA -3′
DoCSLA6F	5′- GCTTATGGAGATTATAAGAAACAA -3′
DoCSLA6R	5′- TCCCAAATCATAGAACCCACAGAA -3′
AtUBQ10F	5′-GATCTTTGCCGGAAAACAATTGGAGGATGGT-3′
AtUBQ10R	5′-CGACTTGTCATTAGAAAGAAAGAGATAACAGG-3′

Gene-specific primers for semi-quantitative RT-PCR were designed by Primer 5.0.
